# The E3 ubiquitin ligase RNF135 regulates the tumorigenesis activity of tongue cancer SCC25 cells

**DOI:** 10.1002/cam4.832

**Published:** 2016-10-05

**Authors:** Jian Jin, liya Zhao, Zubing Li

**Affiliations:** ^1^ The State Key Laboratory Breeding Base of Basic Science of Stomatology (Hubei‐MOST) & Key Laboratory of Oral Biomedicine Ministry of EducationSchool & Hospital of StomatologyWuhan UniversityWuhan430079China; ^2^ Department of Oral and Maxillofacial Trauma and Plastic Aesthetic SurgerySchool & Hospital of StomatologyWuhan UniversityWuhan430079China

**Keywords:** Cell proliferation, E3 ubiquitin ligase, invasion, RNF135, SCC25 cell

## Abstract

Several E3 ubiquitin ligases have been confirmed that they are related to the tumorigenesis. This study aims to find the tongue cancer‐related E3 ubiquitin ligase. The E3 ubiquitin ligase library was screened. The effect of candidate molecule on tongue cancer was validated through cell viability, cell proliferation, colony formation, invasive assay in vitro, and the xenograft model in vivo. The E3 ubiquitin ligase RNF135 significantly promoted the expression of PTEN and TP53 in SCC25 cells. The overexpression of RNF135 inhibited the viability, proliferation, and invasion of SCC25 cells. Knockdown of RNF135 had the opposite effects. Furthermore, RNF135 regulates the tumorigenesis activity of SCC25 cells in vivo. Our results demonstrated that RNF135 had the potential to affect the development of the tongue cancer in vitro. The further in vivo study is helpful to fully understand the role of it.

## Introduction

The global burden caused by cancer is growing at an alarming pace and emphasizes the need for urgent implementation of effectual prevention strategies. Oral cancer is one of the most burning types. It ranks among the 10 most common types of cancer around the world. The 5‐year relative survival rate of oral cancer patients is improving due to the improvement of treatment, but it is far from satisfaction 1. Tongue cancer is the most common type of the oral cancer. Male is more vulnerable than female. The exact pathogenesis of it has not yet been fully understood. Environment factors, genetics, and immune status were believed to be related to the tumorigenesis of the tongue cancer.

Ubiquitin is a highly conserved small molecule protein expressed in eukaryotic cells. It is composed of 76 amino acids. With the help of related enzymes, ubiquitin can bind and ubiquitinate the target protein, which modulates the activity or mediates the degradation of target proteins.

The catalytic process which guides ubiquitin binding to the target protein takes three steps 2. Three kinds of enzymes, E1, E2, and E3 ubiquitin ligase, are requisite in this process. There are two E1 ubiquitin ligases, UbE1 and UBE1L2; 40 E2 ubiquitin ligases and more than 600 E3 ubiquitin ligases in mammalian cells 3.

The dysregulation of E3 ubiquitin ligases as well as their ubiquitin substrates are often associated with human diseases, including cancer 4, 5, 6. E3 ubiquitin ligases are considered as the second most widespread cancer‐related functional family 7. The cancer‐associated proteins, including tumor suppressors, can be degraded by the ubiquitination system. Several cancer, like prostate cancer, breast cancer, colon cancer, ovarian cancer, and leukemia, have been confirmed to be related to the abnormal of E3 ubiquitin ligases. In order to find the tongue cancer‐related E3 ubiquitin ligase, we performed a screening assay of an E3 ubiquitin ligase library and validated the role of candidate protein in the tongue cancer cell lines.

## Materials and Methods

### Ethical approval

This study was approved by the Institutional Review Board (IRB) of Hospital and School of Stomatology, Wuhan University.

### Cell culture

Tongue cancer cell lines SCC25, purchased from American Type Culture Collection, were maintained in Dulbecco's modified Eagle's medium (DMEM) supplemented with 10% fetal bovine serum (FBS), 100 U/mL penicillin, 100 ug/mL streptomycin, and 400 ng/mL hydrocortisone at 37°C with 5% CO_2_. All media for cell culture were purchased from Gibco (Invitrogen, Calsbad, CA).

### E3 ubiquitin ligase screening

The cDNA expression clones encoding ubiquitin‐related enzymes were obtained from the Ubiquitin‐GFC Transfection Array. The array which included 264 genes was purchased from Origene (Cat. #UBGB19601). The clones were transfected into SCC25 cells with lipofectamine 2000. The expression of PTEN and TP53 was detected to screen the candidate molecule.

### Constructs

The mammalian plasmids used were as follows: pcDNA3.1 as vehicle control, pcDNA3.1‐RNF135 was constructed by standard molecular biology techniques.

### RNAi

Double‐stranded oligonucleotides corresponding to the target sequences were cloned into the pSuper.Retro‐RNAi plasmid (Oligoengine).

### Real‐Time PCR

Total RNA was isolated for real‐time PCR analysis to measure the mRNA levels of the indicated genes. Data shown were the relative abundance of the indicated mRNA normalized to that of *GAPDH*.

### Cell proliferation

SCC25 cells (6 × 10^4^) were seeded on the 12‐well plates. RNF135 was transfected with lipofectamine 2000 12 h later. Cell number was calculated at day 2.

### Cell viability

Five thousand SCC25 cells were seeded on the 96‐well plates. RNF135 was transfected with lipofectamine 2000 12 h later. Cell viability was estimated by 3‐ (4,5‐dimethylthiazol‐2‐yl)‐2,5‐diphenyltetrazolium bromide (MTT) metabolism using commercially available Vybrand! MTT Cell Proliferation Assay Kit (Molecular Probes, Invitrogen, Calsbad, CA) at day 2, 4, and 6.

### Colony formation assay

Ten thousand SCC25 cells were plated in 10 cm^2^ cell dish for 18 days. The culture medium was changed every 3 days. Viable colonies were scored with crystal violet staining.

### Cell invasion ability

The analysis of cell invasion ability was performed with 12‐well transwell plates (8.0‐*μ*m pore filters) (Corning, NY). After coating with matrigel basement membrane matrix, 1 × 10^5^ cells were seeded into the upper chamber in a serum‐free DMEM medium. The bottom chamber was filled with DMEM medium including 10% FBS. Then, it was incubated for 24 h at 37^°^C. Then, cells were fixed with 95% ethanol for 20 min and stained with crystal violet for 20 min. The invasive cells on the lower side of the filter were viewed under a light microscope.

### Immunoprecipitation and immunoblotting analysis

Cells (6 × 10^6^) were lysed in l mL Nonidet P‐40 lysis buffer. For each immunoprecipitation, 0.4 mL cell lysate was incubated with 0.5 *μ*g of the anti‐Flag antibody and 30 *μ*L of 50% (vol/vol) slurry of GammaBind G Plus Sepharose (Amersham Biosciences) at 4°C for 3 h. The Sepharose beads were washed three times with 1 mL of lysis buffer containing 0.5 mol/L NaCl. The precipitates were fractionated by SDS/PAGE electrophoresis and subsequent immunoblotting analysis was performed with the anti‐HA antibody.

### Xenograft models

A total of eight 8‐week‐old male athymic immunodeficient Balb/c nude mice were recruited in the metastasis lung tumor assay. The RNF135 stable knockdown SCC25 cells (5 × 10^7^) were injected through tail vein. The lungs were harvested 10 weeks later and fixed in 4% PFA for histological analysis.

### Statistical analysis

The results were graphically depicted as the mean ± the standard deviation (SD). Two‐tailed t‐test and one‐way analysis of variance (ANOVA) were performed (SPSS 13.0 for Windows, SPSS, Chicago, IL) to detect statistically significant differences. *P* < 0.05 was considered statistically significant.

## Results

### RNF135 as a candidate tongue cancer‐related E3 ubiquitin ligase

**Figure 1:**
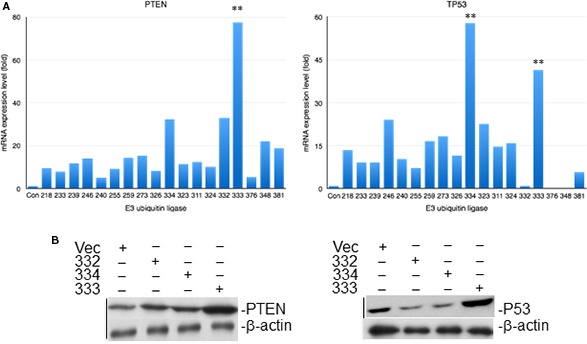
The screening of candidate tongue cancer‐related E3 ubiquitin ligase. The E3 ubiquitin ligase which was numbered sequentially was cotransfected to SCC25 cells. The 333 is RNF135. It greatly promoted the expression of PTEN and P53 in both mRNA (A) and protein level (B). (**:*P* < 0.01).

After transfection with different E3 ubiquitin ligase, the expression of tumor suppressor gene *PTEN* and *TP53* in both mRNA and protein level was used to select the sensitive molecule. RNF135 aroused our interest because it significantly promoted the expression of both PTEN and TP53 in SCC25 cells (Fig. 1).

### RNF135 inhibits the proliferation and colony formation of SCC25 cells

**Figure 2:**
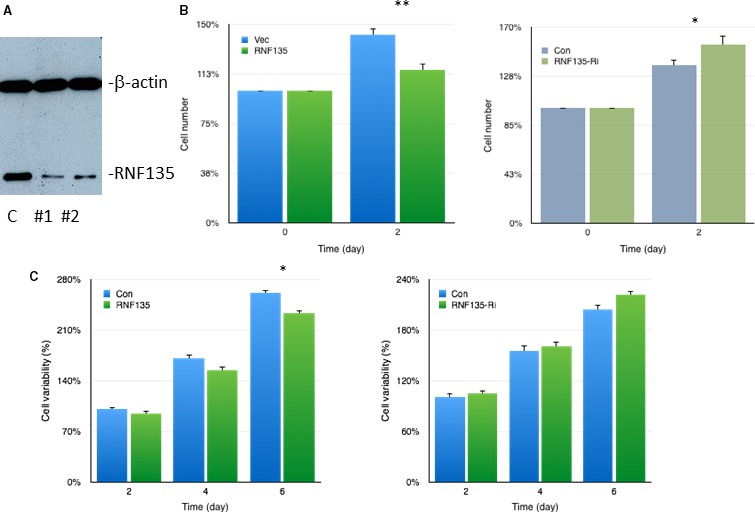
RNF135 regulated the cell proliferation and viability of SCC25 cells. (A) The knockdown efficient of RNF135 RNAi. Two RNAi of RNF135 was constructed. The #1 was used in the following study according to its efficient. C:control. (B) The growth of SCC25 decreased by 20% with the overexpression of RNF135. (C) RNF135 regulated the viability of SCC25 cells. (*:*P* < 0.05 **:*P*<0.01.)

**Figure 3:**
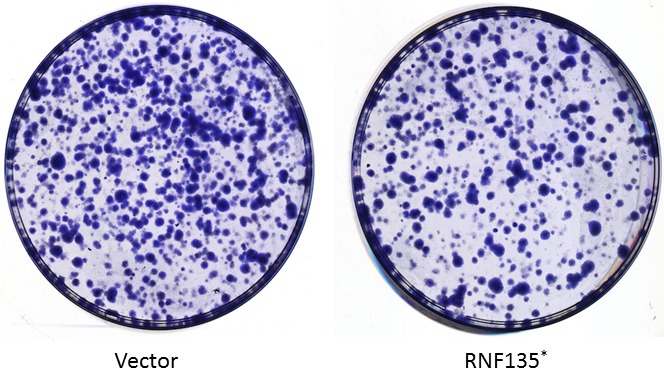
The effect of RNF135 on colony formation ability of single SCC25 cell. Compared to the control group, overexpression of RNF135 significantly inhibited the colony formation (*:*P* < 0.05).

In order to validate the results of screening assay, we investigated the biological activity changes of SCC25 cells affected by RNF135. Firstly, we determined the effect of RNF135 on tongue cancer cell's proliferation. The overexpression of RNF135 inhibited the proliferation of SCC25 cells by 20% compared to the control group at day 2. Then, we detected the cell viability changes. It also decreased at the three detection times. Next, the cell growth changes were evaluated under the inhibition of RNF135 (Fig. 2). The results suggested that the number of SCC25 cells had a significant increase with the knockdown of RNF135 by RNAi. The colony formation ability of single SCC25 cell was also determined. It was evidently inhibited when RNF135 was overexpressed (Fig. 3). Based on these results, we concluded that RNF135 had an influence on the growth of SCC25 cells.

### RNF135 inhibits the invasion of SCC25

**Figure 4:**
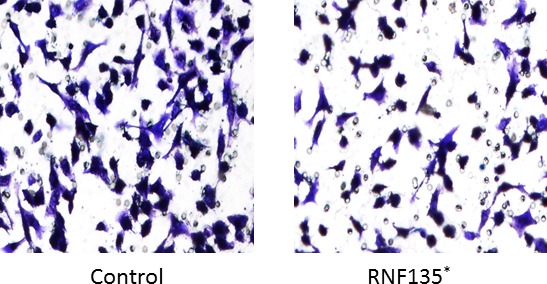
The overexpression of RNF135 inhibited the invasive ability of SCC25 cells (*:*P* < 0.05).

The invasive ability is one of the main characters of tumor cell. We studied the invasion of SCC25 using transwell assay. The overexpression of SCC25 greatly inhibited the aggressive features. Compared to the control group, only half of the SCC25 cells transmitted through the matrigel and the well (Fig. 4).

### RNF135 regulates the AKT signaling

**Figure 5:**
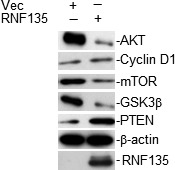
RNF135 was involved in the AKT signaling pathway.

As an E3 ubiquitin ligase, we hypothesize that RNF135 was involved in tumorigenesis by ubiquitinating the substrates. Then, we attempted to analyze the interacted protein of RNF135 through immunoprecipitates. We currently did not find the substrate of RNF135 and only concluded that the key molecules of PTEN signaling were excluded. But the expression changes of AKT as well as its downstream molecules were noticed (Fig. 5).

### RNF135 regulates the tumorigenesis activity of SCC25 cells in vivo

**Figure 6:**
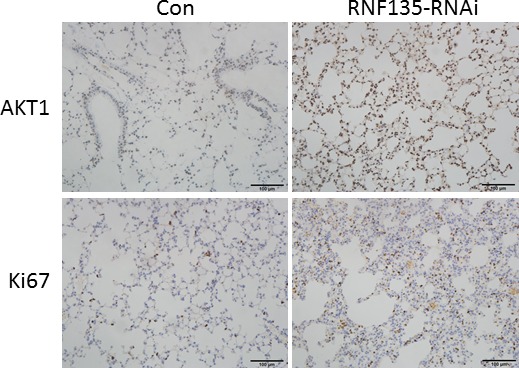
Knockdown of RNF135 promotes the metastatic tumor growth in vivo. The expression of Ki67 and AKT was increased in metastatic lung cancer specimens of mice which was injected with the RNF135 knockdown SCC25 cells. Scale bar = 100 *μ*m.

To further elucidate the physiological role of RNF135 in regulating the tumorigenesis activity of SCC25 cells in vivo, we established the metastasis lung cancer model through injecting the RNF135 stable knockdown SCC25 cells into the nude mice through tail vein. During the following 10‐week observation period, all mice survived. Ki67 is a biomarker for cell proliferation and prognosis. We performed immunohistochemistry staining experiments and detected the expression of Ki67 in cancer specimens. As shown in Figure 6, the expression of Ki67 was strongly increased in the metastatic tumors harvested in the RNF135 knockdown group. Furthermore, we observed that the expression of AKT was also promoted in the RNF135 knockdown group.

## Discussion

Our experience of clinical practice suggested that there was an urgent need to find new diagnosis and treatment biomarkers for tongue cancer. The reasons are obvious. Firstly, tongue muscles are often invaded by the lesion. As a result, the movement of the tongue is restricted. Patients have great troubles in speaking, eating, and swallowing. Secondly, the metastasis frequency is higher in tongue cancer because the tongue body is rich in lymphatic vessels as well as has frequent mechanical movements. The distant metastasis was also common, especially to the lungs. Next, more than half oral cancer cases were diagnosed when the tumors had become locally advanced 8. These patients did not response well to the current treatment effect.

Recently, with the development of molecular genetic analysis, the successful application of specific molecular targets for cancers, like lung cancer, has greatly improved the therapeutic outcome 9, 10. This strategy can also be applied in tongue cancer. This issue had already drawn the attention of several scientists 11. We focused on the roles of E3 ubiquitin ligase in this study.

Based on the structure character, E3 can be divided into four categories, E3 ubiquitin ligase with HECT (homologous to E6‐associated protein carboxyl terminus) domain, with ring (really interesting new gene) domain, with U‐box domain, and with RBR (RING‐in‐between‐RING) domain 12. The E3 ubiquitin ligase with ring domain is the most classic one. The ubiquitin chain connection of them which need to rely on the catalytic specificity of E2 ubiquitin‐binding enzyme is not selective 13. HECT E3 ubiquitin ligase which includes Nedd4, Smurf2, rsp5, and E6AP has ubiquitin chain catalytic specificity 14, 15. Several E3 ubiquitin ligases were involved in the oncogenesis of oral cancer, lung cancer, and breast cancer. In order to find the sensitive E3 ubiquitin ligases of tongue cancer, we screened a human library. RNF135 was selected, for it strongly promoted the expression of both *PTEN* and *TP53* in mRNA and protein level. It is an E3 ubiquitin ligase with RING domain. The role of it in cellular biology is still unknown. Our results suggested that it showed evident effect on both cell proliferation and viability of tongue cancer cells. The invasion of SCC25 cells was also decreased with the overexpression of RNF135. We speculated that it might be involved in the development of tongue cancer. The ubiquitination of RNF135 on target protein might be the possible mechanism. We tried to find the substrates of RNF135 on PTEN signaling using immunoprecipitation. Although no interaction was detected, we found the expression of key protein in PTEN signaling greatly changed. These data suggested RNF135 was involved in PTEN signaling. We will further analyze the substrate of it in the future.

In a word, we reported a tongue cancer‐sensitive E3 ubiquitin ligase. Our results demonstrated that it had the potential to inhibit the development of the tumor in vitro. The further in vivo study is helpful to fully understand the role of it in tongue cancer.

## Conflict of Interest

None declared.
